# Definite hereditary hemorrhagic telangiectasia in a 60-year-old black Kenyan woman: a case report

**DOI:** 10.1186/s13256-016-0909-4

**Published:** 2016-05-25

**Authors:** Joan Chepkorir Kiyeng, Abraham Siika, Cornelius Koech, Gerald S. Bloomfield

**Affiliations:** 1 Department of Internal Medicine, College of Health Sciences, School of Medicine, Moi University, P.O Box 4606, Eldoret, Kenya; 2 Division of Cardiology, Duke University Medical Center, 2400 Pratt Street, Durham, NC 27705 USA

**Keywords:** Arteriovenous malformations, Epistaxis, Hereditary hemorrhagic telangiectasia

## Abstract

**Background:**

Hereditary hemorrhagic telangiectasia is a rare autosomal dominant inherited disease characterized by vascular dysplasia. To the best of our knowledge, we report the first case in the literature of definite hereditary hemorrhagic telangiectasia diagnosed in western Kenya, a resource-limited setting with limited treatment options.

**Case presentation:**

A 60-year-old black Kenyan woman was admitted 1 year ago to a hospital in western Kenya with an 11-year history of recurrent spontaneous epistaxis. Her physical examination revealed that she had telangiectasias on the tongue and hard palate, severe pallor, and hepatomegaly. A chest radiograph revealed right middle lobe opacity. After a positive saline contrast echocardiography, she underwent contrast-enhanced chest computed tomography, which revealed a large pulmonary arteriovenous malformation and multiple hepatic arteriovenous malformations. Therefore, she fulfilled criteria for definite hereditary hemorrhagic telangiectasia. She was managed with nasal packing, tranexamic acid, oral ferrous sulfate, and blood transfusions, as other treatment options were unavailable in this setting.

**Conclusions:**

This rare case of hereditary hemorrhagic telangiectasia demonstrates that it occurs in an African population and that diagnostic challenges in resource-limited settings can be surmounted. Treatment options remain limited in these settings.

**Electronic supplementary material:**

The online version of this article (doi:10.1186/s13256-016-0909-4) contains supplementary material, which is available to authorized users.

## Background

Hereditary hemorrhagic telangiectasia (HHT) is a rare disease worldwide. It is an autosomal dominant inherited disease that causes vascular dysplasia mainly affecting mucocutaneous and visceral organs. Only one case of possible HHT has been described previously in sub-Saharan Africa (SSA) [1]. Clinical presentations of HHT are age-related and variable, with epistaxis being the most common presenting symptom [2, 3]. Diagnosis and management of HHT are challenging in areas with scarce resources such as Kenya. This case report and literature review highlights these challenges.

## Case presentation

We present a case of a 60-year-old Kenyan woman who was admitted 1 year ago to a hospital in western Kenya with complaints of epistaxis and right-sided chest pain for the preceding 3 days. She reported an 11-year history of recurrent spontaneous epistaxis that had worsened 3 days before admission. Apart from one episode of postpartum hemorrhage (PPH) in the past that had required blood transfusion, she had no other history of bleeding tendencies. Her right-sided chest pain was pricking in nature and radiated to her upper back. She had associated shortness of breath but no cough or platypnea. She also complained of severe headache that she described as global, persistent, and not worsened by light but associated with dizziness. She had never been on any medications for the epistaxis.

She had two previous admissions: one 17 years ago for PPH that required blood transfusion and another in early childhood for malaria. She is a widow, mother of five children, and a first-born in a family of seven. Two of her sisters had histories of mild recurrent epistaxis. She had a history of occasional alcohol use but had stopped 4 years before admission. She had no history of tobacco use.

Her physical examination revealed a middle-aged woman in fair general condition with severe pallor and a tinge of jaundice. She had no cyanosis or lymphadenopathy. Her vital signs were blood pressure of 95/60 mmHg, pulse rate of 100 beats/minute, respiratory rate of 22 breaths/minute, and axillary temperature of 37 °C. Her oxygen saturation by pulse oximetry was 95 % on room air, which dropped to 92 % 5 minutes after changing positions from supine to upright.

**Fig. 1:**
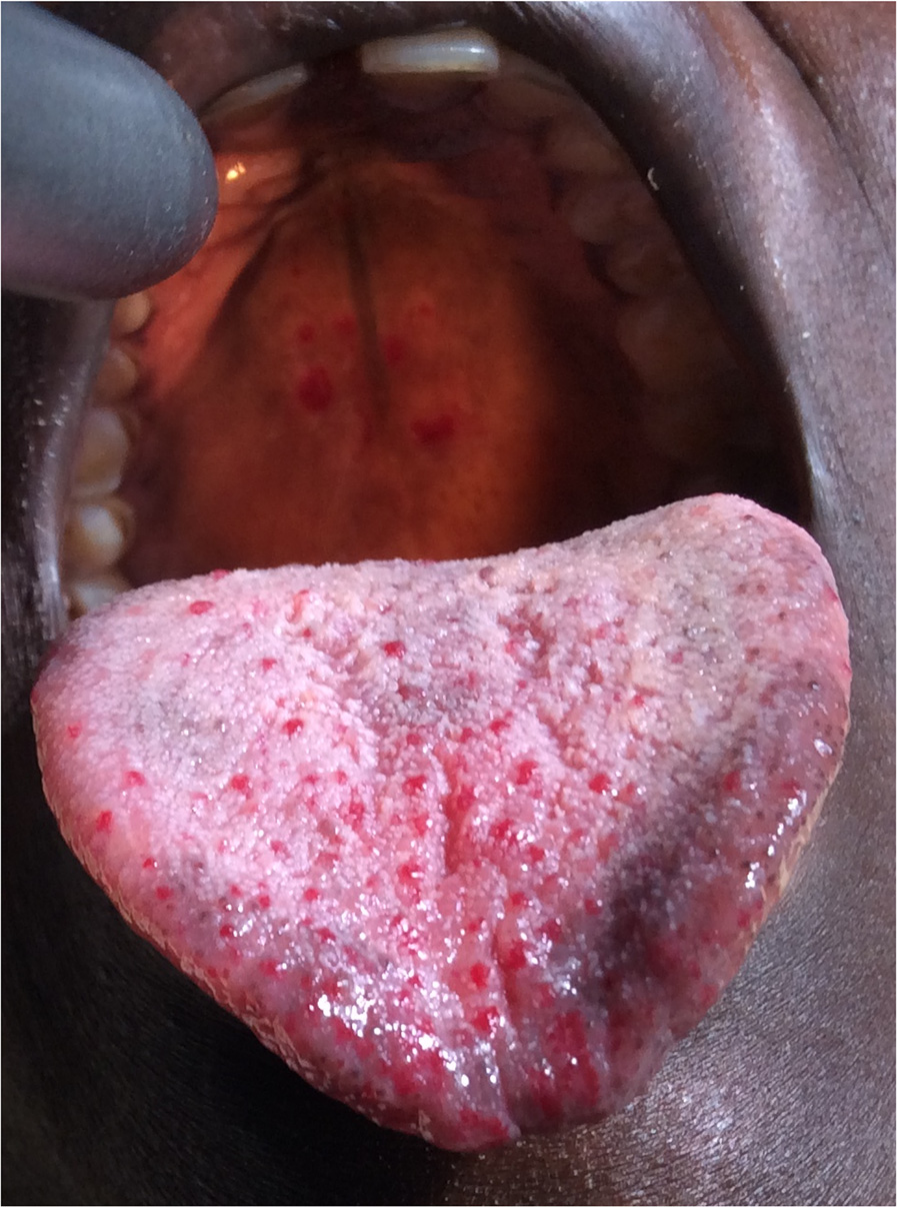
Telangiectasias on the tongue and hard palate. A photograph of the patient’s oral cavity showing multiple, guttate-like, erythematous lesions with a tendency to coalesce on the edges of the tongue, as well as a few grouped erythematous, well-demarcated lesions on the hard palate. The lesions were nontender and blanched upon pressure

Her oral examination revealed multiple, guttate-like, erythematous blanching lesions with a tendency to coalesce on the edges of the tongue, as well as a few grouped erythematous, well-demarcated, blanching lesions on the hard palate (Fig. 1).

Her cardiovascular examination was notable for mild tachycardia, a bounding pulse, and a hemic murmur. She had hepatomegaly of 7 cm below the costal margin with a liver span of 17 cm and a bruit on auscultation. The rest of her systemic examination was unremarkable.

**Fig. 2:**
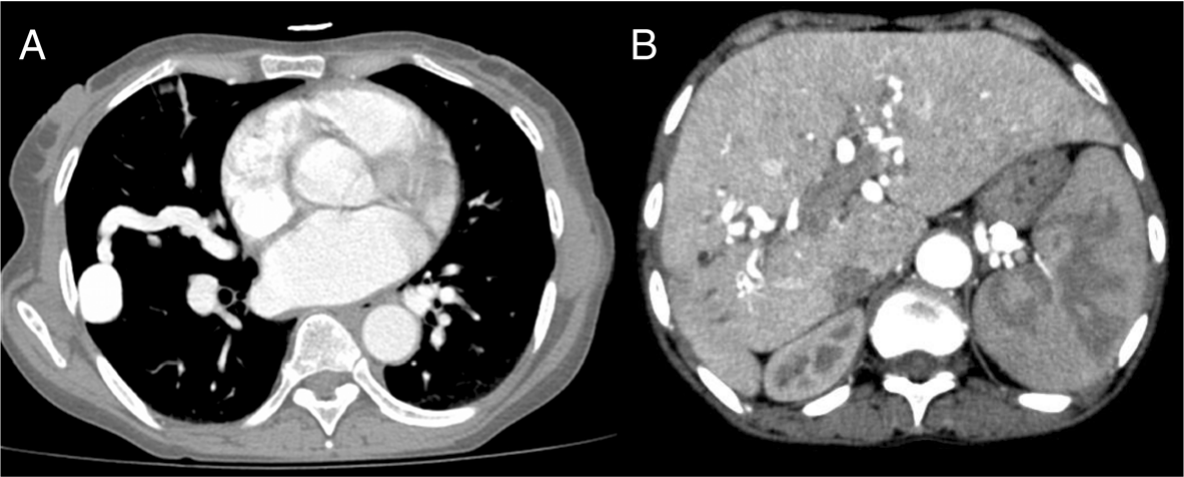
Computed tomography of the chest demonstrating a large pulmonary arteriovenous malformation (**a**) and multiple hepatic arteriovenous malformations (**b**). Contrast-enhanced computed tomographic scans show (**a**) a dilated vessel in the right midperipheral lung region creating a pool of contrast, as well as a large draining vein into the right pulmonary vein suggestive of an arteriovenous malformation, and also show (**b**) multiple intrahepatic tortuous vessels enhancing on arterial phase suggestive of intrahepatic arteriovenous malformations and peripheral smaller ones with early venous phase displaying early filling of the hepatic veins. The splenic hilum also shows tortuous vessels suggestive of splenic hilar arteriovenous malformations

Her laboratory investigations revealed normal activated partial thromboplastin time, prothrombin time, international normalized ratio, and bleeding time. Her complete blood count showed hemoglobin of 4.1 g/dl, mean corpuscular volume of 60.6 fl, a normal white blood cell count, and slight thrombocytosis of 471 × 10^3^/μl. Her peripheral blood smear showed a decreased red blood cell count with moderate microcytosis and marked hypochromasia, suggestive of iron deficiency anemia. She had slightly elevated alkaline phosphatase and total bilirubin levels. A chest radiograph demonstrated opacity in the right midzone, and a transthoracic contrast echocardiography (TTCE) with agitated saline confirmed the presence of a right-to-left shunt (Additional file 1: Video 1). Considering either a large atrium-level or pulmonary shunt, we then performed contrast-enhanced chest computed tomography (CT), which revealed a large pulmonary arteriovenous malformation (AVM) and multiple hepatic AVMs (Fig. 2).

At presentation, we considered inherited coagulopathies such as von Willebrand disease, given the histories of her two siblings, chronic liver disease, and platelets disorders as possible causes of epistaxis. These were ruled out by normal coagulation studies and bleeding time, however.

The diagnosis of HHT is based on the international consensus diagnostic criteria (the Curacao diagnostic criteria), which use clinical findings of epistaxis, mucocutaneous telangiectasias, visceral vascular malformation, and positive family history (Table 1) [4]. Our patient was diagnosed with definite HHT, as she fulfilled three criteria. Although she had reported that two her siblings had histories of mild epistaxis, this criterion was not considered in this case, as the siblings’ diagnoses were not confirmed to be HHT.

**Table 1:** Diagnostic criteria for hereditary hemorrhagic telangiectasia (Curacao criteria)

Criteria	Characteristics
Epistaxis (nosebleeds)	Spontaneous and recurrent epistaxis
Mucocutaneous telangiectasias	Multiple telangiectasias at characteristic sites (for example, lips, tongue, nose and hands)
Arteriovenous malformations	Involving visceral organs, including the pulmonary, gastrointestinal, hepatic, and cerebral vasculature
Family history of hereditary hemorrhagic telangiectasia	A first-degree relative with hereditary hemorrhagic telangiectasia

The hereditary hemorrhagic telangiectasia diagnosis is classified as definite if three or four criteria are present; possible/suspected if two criteria are present; and unlikely if fewer than two criteria are present [4]

Diagnosis of this rare disease is a challenge in resource-limited settings due to its variability in presentation and lack of clear diagnostic modalities. As shown in our patient, late diagnosis is due to many factors, including low clinical suspicion among clinicians, difficulties in accessing health care by the patient, and economic costs associated with further imaging. The costs of these investigations were waived for our patient.

The patient’s epistaxis was controlled by repeated nasal packing. She also received 1 g of tranexamic acid intramuscularly, which was continued orally at 500 mg three times per day for 5 days. Her epistaxis stopped 5 days after admission. She also received a transfusion of 6 U of whole blood and was put on oral ferrous sulfate 325 mg three times daily. Her repeat complete blood count showed hemoglobin of 12 g/dl 10 days after admission. She was discharged to home on oral ferrous sulfate with follow-up in our clinic, as other clinical management procedures were not available. One year after her discharge, our patient was still experiencing intermittent epistaxis and remained on iron therapy.

## Discussion

Hereditary hemorrhagic telangiectasia (HHT), also called *Rendu-Osler-Weber syndrome*, is a rare autosomal dominant inherited systemic disease. It is characterized by telangiectasias of the skin and mucous membranes as well as large AVMs mainly in pulmonary, hepatic, and cerebral circulation. Globally, the prevalence varies between 1 in 5,000 and 1 in 10,000 [5]. Notably, in SSA, only one case of possible HHT, in a black African male in Nairobi, Kenya, has been described [1].

Clinical manifestations of HHT vary with age, with spontaneous recurrent epistaxis being the most common, affecting 90 % of the patients by age 21 years [2, 3]. Our patient developed epistaxis at the age of 49 years, which was later than would have been expected. Most patients present with severe iron deficiency anemia requiring blood transfusion and iron supplementation. Although our patient was managed with repeated nasal packing and tranexamic acid, in a resource-sufficient setting she could have benefited from other options, such as laser ablation, arterial ligation, septodermoplasty, or systemic therapies [6–8].

The initial appearance of our patient’s oral telangiectatic lesions is unknown, and she never reported any bleeding. Telangiectasias are more common with increasing age, rarely bleed, and affect 70 % of patients by age 30 years [2]. Recurrent gastrointestinal (GI) bleeding occurs in up to 30 % of patients as a result of telangiectasias [2]. Our patient had no history of GI symptoms.

Visceral AVMs, which are predominantly asymptomatic, occur in the pulmonary (50 %), hepatic (30 %), cerebral (10 %), and, rarely, spinal vasculature (1 %) [5]. Pulmonary AVMs can rarely present with paradoxical systemic embolization, leading to ischemic stroke or brain abscess [9]. Our patient had both pulmonary and hepatic AVMs. She was not screened for cerebral AVMs, but there are reports in the literature of these AVMs as being multiple and with a low risk of hemorrhage [10]. The headache described by our patient was unlikely to have been migraine with aura, which has been reported to occur in some patients [11]. Our patient had no clinical signs of central nervous system disease on examination.

Chest pain and dyspnea in our patient were likely due to hypoxemia resulting from a secondary pulmonary shunt, compounded by severe anemia. During saline TTCE, the microbubbles appeared during the fourth beat in the left atrium, but there was poor visualization of the shunt origin. CT scans confirmed the presence of a large right pulmonary AVM with a feeding artery of 6.35 mm. The feeding artery met the criterion for transcatheter embolotherapy, which considers a ≥3 mm as a threshold above which to warrant this procedure [12]. It was not possible to carry out this intervention in the local hospital; however, in accordance with the international guidelines recommendations, the patient was advised on the use of antibiotic prophylaxis before high-risk procedures to prevent brain abscess [7].

Hepatic AVMs can lead to complications such as high-output heart failure and portal hypertension-associated complications, and this can be managed with medical therapy. However, if this therapy fails, then invasive treatment with embolization and liver transplant can be used to eliminate the hepatic AVMs [13].

## Conclusions

This rare case of a patient with HHT demonstrates that it occurs in our SSA population and that diagnostic challenges in resource-limited settings can be surmounted. However, treatment remains limited in these settings.

## Additional file

Additional file 1: Video 1. Video recording of saline transthoracic contrast echocardiography. A video recording of transthoracic contrast echocardiography (TTCE) showing the presence of right-to-left shunt after agitated saline was injected into a peripheral vein. The saline contrast appears on the left side of the heart during the fourth cardiac cycle. (MOV 6475 kb)

## Abbreviations

AVM: arteriovenous malformation
CT: computed tomography
GI: gastrointestinal
HHT: hereditary hemorrhagic telangiectasia
PPH: postpartum hemorrhage
SSA: sub-Saharan Africa
TTCE: transthoracic contrast echocardiography
